# Factors Affecting COVID-19 Vaccine Acceptance among Pregnant Women: A Cross Sectional Study from Abha City, Saudi Arabia

**DOI:** 10.3390/vaccines11091463

**Published:** 2023-09-07

**Authors:** Asma Saad Habbash, Aesha Farheen Siddiqui

**Affiliations:** Department of Family and Community Medicine, College of Medicine, King Khalid University, Abha 62529, Saudi Arabia

**Keywords:** COVID-19, pregnancy, vaccine acceptance, Saudi Arabia

## Abstract

**Background**: Pregnant women can get infected with COVID-19 with serious sequelae to them and their fetus. Concerns about COVID-19 vaccination safety to mothers and babies, and doubts about its effectiveness, have hindered vaccine acceptance throughout the COVID-19 crisis. The objective of the current investigation was to estimate COVID-19 acceptance rates among pregnant women in Abha city, Aseer region, Saudi Arabia, and determine its clinical and demographic correlates. **Method**: Descriptive questionnaire-based cross-sectional survey of a sample of pregnant women attending regular antenatal care services in Abha. We used backward stepwise multiple logistic regression analysis to evaluate the predictability of vaccine acceptance in terms of baseline clinical and demographic factors. **Results**: The survey included 572 pregnant women. The prevalence of acceptance of COVID-19 vaccine was high (93.7%; 95%CI: 91.7–95.7%). University graduates and women with a later gestational age were more likely to accept vaccination (OR = 6.120, *p* = 0.009), (t = 2.163, *p* = 0.036), respectively. Confidence in vaccine safety was associated with better acceptance (OR = 3.431, *p* = 0.001). **Conclusions**: The acceptance rate for vaccination among pregnant women in Abha, Saudi Arabia, is higher compared to international rate. However, our results indicate that confidence in vaccine safety was associated with better acceptance. Hence, vaccine safety was the overarching predictor for harboring positive attitudes towards it. Public health policies should capitalize on such positive attitudes and aim for total coverage of pregnant women with COVID-19 vaccination including booster dosages.

## 1. Introduction

Over three years after it first started in December 2019 in China, the Severe Acute Respiratory Syndrome Coronavirus 2 (SARS-CoV-2) or COVID-19 pandemic is still continuing to affect the world. Over 690 million confirmed cases and over 6.8 million deaths have been reported globally [1] as of 29 June 2023. After it first started, random mutations of SARS-CoV-2’s genome led to genetic variants. The Alpha and the Delta variants were observed to be more transmissible. There are many variants of severe acute respiratory syndrome coronavirus 2 (SARS-CoV-2), some of which have particular importance due to their potential for increased transmissibility, increased virulence, or the reduced effectiveness of vaccines against them. These variants contribute to the continuation of the COVID-19 pandemic [2]. As of August 2023, Omicron is designated as a circulating variant of concern by the World Health Organization, and a newest variant BA.2.86 (pirola) is starting to cause concern in different parts of the world.

In addition to substantial mortality and morbidity, the COVID-19 crisis posed significant public health and mental health challenges internationally [3,4] as well as in Saudi Arabia [5]. At the start of the pandemic, virus transmission and its resulting morbidity and mortality were reduced through an array of measures like individuals practicing social distancing, using facemasks, practicing frequent hand hygiene, and restricting interpersonal contact. Aside from these preventive measures, widespread testing was promoted to identify individuals infected with the virus and curfews and closures were put in place by governments, including school and workplace closures, bans on public gatherings, and travel restrictions [6]. Despite such measures, the COVID-19 pandemic kept raging for 2020 and most part of 2021. Because of the severe morbidity and mortality entailed by this disease, the best option, that of a vaccine, was followed on a war footing. To conquer the virus in this war, scientific and pharmaceutical institutions worldwide strived hard for the development of safe and effective COVID-19 vaccines [7]. If we examine in short the timeline of the vaccine development, we find that the genetic sequence of the virus was shared by Chinese researchers, enabling scientists worldwide to begin working on potential vaccines, within one month of the first case reported from Wuhan, China. The clinical trials for COVID-19 vaccines began in various countries, including the United States, China, and Europe in March 2020 and highly effective COVID-19 vaccines based on mRNA technology were announced in November 2020 by Pfizer-BioNTech and Moderna. Soon after, in December 2020, several countries, including the United States, United Kingdom, and Canada, granted emergency use authorization for the Pfizer-BioNTech and Moderna vaccines. Throughout 2021, additional COVID-19 vaccines, such as those developed by AstraZeneca, Johnson and Johnson, and Novavax, received regulatory approvals and were rolled out globally. Vaccine distribution efforts continued worldwide, with many countries implementing vaccination campaigns to combat the spread of COVID-19 [8].

Like most vaccines, the COVID-19 vaccines stimulate the immune system to recognize and fight specific pathogens, and in this case, the SARS-CoV-2 virus. Several COVID-19 vaccines have been authorized for emergency use or approved by regulatory authorities in different countries. These vaccines have been the subject of extensive clinical trials to evaluate their efficacy, tolerability, and ability to provide COVID-19 protection. Research studies recruited hundreds of individuals and, followed strict protocols in order to collect data on the efficacy, adverse effects, and long-term protection of the vaccines. Regulatory agencies examined the results of these experiments before authorizing or approving their use [6,7].

Though the vaccines were developed at a remarkable speed, for them to be efficacious in controlling the COVID-19 crisis, there is a requirement for substantial rate of vaccine acceptance. The least estimate required for control of a COVID-19 pandemic is a 75% acceptance rate [9]. Strategies should be standardized to enhance vaccine acceptance and fight vaccine hesitancy [10]. For effective curbing of the current pandemic, all population groups, including pregnant women, must be vaccinated [11].

Pregnant women form one of the vulnerable population groups. Pregnant women are not as susceptible to COVID-19 as everybody else, with increased risk of severe disease [12]. The rate of symptomatic COVID-19 among pregnant women is estimated at a 45%, with 8.5% risk for serious illness requiring mechanical ventilation and 1% mortality [13]. Preventative measures remain the first line to defend pregnant women and their offspring from this potentially fatal disease. Hence, pregnant women, given the increased risk of morbidity and mortality related to COVID-19, should be offered, and encouraged to take, the vaccine [14].

However, the data on safety and efficacy of COVID-19 vaccine on pregnant women is extremely limited. Vaccine advocates campaigned for pregnant women to be included in vaccine trials across the world given the importance of protection against this potentially fatal disease [15,16]

A review of the literature reveals in a trans-continental survey that included over 17,000 pregnant women among 16 countries an estimated acceptance rate of 52% (substantially lower than the 73.4% figure for their non-pregnant counterparts). Higher vaccine acceptance was noted in Asian and Latin American countries. Low vaccine acceptance was notable in Russia, the United States, and Australia [9]. A recent Turkish investigation [17] was designed to determine COVID-19-related vaccine acceptance and hesitancy with a focus on pregnant women. They interviewed 300 pregnant women with a questionnaire that included vaccination history and acceptance of and attitude toward future COVID-19 vaccination. They found the acceptance rate to be low (namely, 37%). Better acceptance was noted for those pregnant women in their first trimester. Vaccine acceptance has shown to be associated with being concerned about COVID-19, better confidence in vaccine safety and effectiveness, worrying about COVID-19, and trust of public health agencies/health science, in addition to favourable attitudes towards routine vaccines [9]. On the other hand, vaccine hesitancy, which is defined by the WHO as either delayed acceptance or refusal of a vaccine despite its availability, is a major global health threat. The WHO’s Strategic Advisory Group of Experts (SAGE) explains vaccine hesitancy through three main reasons: confidence or worries about the safety and effectiveness of the vaccine; complacency or low self-perceived risk of a vaccine-preventable disease and convenience matters include availability, accessibility, affordability and the widespread reach and understanding of health messages (health literacy) from authorities about the vaccine [18]. Although vaccine hesitancy is not a major public health issue in Saudi Arabia, as it offers 100% free immunization services to all citizens and residents, the scenario with COVID-19 can be expected to be different more so with the pregnant women as it is a new and fatal infectious disease. Thus, the current investigation was designed to estimate the acceptance rate of COVID-19 vaccination and its associated factors among pregnant women in Abha city, Aseer region in Saudi Arabia, at the peak of the epidemic.

## 2. Methodology

### 2.1. Study Design, Location and Study Period

This was a cross-sectional questionnaire-based study on 572 pregnant women attending six primary health care centers in Abha, Saudi Arabia, between March and August 2021.

### 2.2. Sample Size Calculation and Sampling Technique

The study used all pregnant women attendees at the antenatal clinics chosen from the 12 Primary Health Care Centres (PHCCs) in Abha, Aseer region, as a sampling frame. We calculated the sample size using the equation described in [19], n = (Zα/2/e)2 × [p(1 − p)], where Z is the value taken by the standard normal distribution variable Z at α/2 (for α at 0.05, the Z value is 1.96), e is the error margin (assumed 4%), and p is the previous best estimate (52%) [19], to get a desired sample size of 400. The sample was recruited from 6 PHCCs in Abha selected by draw, and participants were recruited by visiting the antenatal clinic and consequently sampling until the desired sample size was achieved. We recruited the sample over and above the required sample size, and a total of 601 responses were received. Twenty nine questionnaires were discarded for incompleteness and our final sample included 572 pregnant women.

### 2.3. Inclusion and Exclusion Criteria

Inclusion criteria for the study was pregnant women over 18 years of age attending primary healthcare centres in Abha, Saudi Arabia, for ante-natal care between March 2021 and August 2021 and were willing to participate. Those refusing to participate were excluded.

### 2.4. Study Tool

A study questionnaire derived from previous studies was used for data collection [9,17]. This questionnaire reported high internal consistency, face validity, and construct validity. The questionnaire consisted of three parts. First, details of demographic data were collected, including age, residence, education, and employment. Second, clinical data were sought in terms of pregnancy stage and history of COVID-19 infection. Third, data pertaining to willingness to getting the vaccine and acceptance were also collected by the research tool.

### 2.5. Independent and Dependent Variables

COVID-19 vaccine acceptance rate was the dependent variable and factors affecting it included demographic data, obstetric data, and COVID-19 related disease experience.

### 2.6. Data Collection and Management

Data was collected by face-to-face interviewing pregnant women during their regular antenatal check-up visits. Data were entered into a Windows 10 Microsoft Excel Sheet and stored on the researcher’s personal computer.

### 2.7. Data Analysis

The demographic variables and background clinical characteristics of patients were presented with numbers and percentages for categorical variables; mean and standard deviation (SD) was used for continuous variables. The effect of socio-demographic factors on COVID-19 acceptance was assessed using Chi square /Fisher’s test for categorical variables and student’s t test for continuous variables. Those factors with a significant relationship (*p* < 0.05) with the dependent variables in the univariate model were included to evaluate the predictability of vaccine acceptance in terms of baseline clinical and demographic factors using regression analysis. Multiple logistic regression was used to assess the relationship between two or more continuous or categorical explanatory variables and a single categorical response variable. The categorical response variable in our study was vaccine acceptance. In univariate analysis, gestational age education and occupation were significant among the background variables; among the COVID-19-disease-related variables, family loss and use of masks was significant and in the COVID-19-vaccine-related variables, vaccine safety, vaccine efficacy, vaccine importance, and vaccine fear were significant. All these factors were entered in a single full model backward stepwise multiple logistic regression model to account for the simultaneous effects of all of them. All the analyses were conducted using R Statistical Software 3.6.0 [20].

### 2.8. Ethical Considerations

The study was conducted in accordance with the Declaration of Helsinki, and approved by the Institutional Review Board of King Khalid University Re-search Ethics Committee approval number (REC#2021-02-02).

## 3. Results

The total number of women included in the study was n = 572. For a detailed account of demographic results see Table 1 below.

The prevalence of acceptance to get COVID-19 vaccine was high (n = 536, 93.7% [95%CI: 91.7% to 95.7%]) among the participating women. Only (n = 36, 6.3%) were the minority to openly declare their opposition for COVID-19 vaccination.

The mean age for the participants was 29.9 years (SD = 6.92 years), ranging between 18 and 45 years of age. Those accepting of COVID-19 vaccination were marginally younger (mean age was 29.9 years) compared to 31 years in those not accepting of COVID-19 vaccine. However, the difference was not statistically significant (t = 1.0498, *p* = 0.2999).

The mean number of offspring for the participants was 2.2 children (SD = 2.1 children), ranging between 0 and 10 kids. Those accepting of COVID-19 vaccination had less children (mean number was 2.2 children, compared to three children in those not accepting of COVID-19 vaccine). This difference was statistically significant (t = 2.07, *p* = 0.044).

In terms of educational qualifications, the majority were university graduates (n = 420, 73.4%) and were the highest category in terms of COVID-19 vaccine acceptance (n = 399, 95%) after women with postgraduate education (n = 12, 100%). The effect of educational background on vaccine acceptance was statistically significant (χ^2^_(5)_ = 19.333, *p* = 0.001).

The mean pregnancy age among the participants was 15.1 weeks (SD = 11.9 weeks), ranging between 0 and 42 weeks. Those accepting of COVID-19 vaccination were at a later gestational age (mean pregnancy age was 15.4 ± 11.7 weeks, compared to 11.6 ± 11.2 weeks in those not accepting of COVID-19 vaccine). This difference was statistically significant (t = 2.16, *p* = 0.036).

Table 2 and Table 3 give a detailed account of the COVID-19-related factors among the participating women and their unadjusted effect on COVID-19 vaccine acceptance. Vaccine acceptance was higher among those with diagnosed COVID-19 compared to those without, 98.2% and 92.6%, respectively. However, this was not statistically significant (χ^2^_(1)_ = 3.73, *p* = 0.053). Women who experienced family loss secondary to COVID-19 reported 100% vaccine acceptance, compared to 92.7% acceptance rate in those who did not (χ^2^_(1)_ = 5.06, *p* = 0.024). Moreover, the more confidence participants had in vaccine safety, the more likely they were to accept it (z = 2.39, *p* = 0.01). Similarly, the more they believed about vaccine importance, the higher was their acceptance rate (z = 2.10, *p* = 0.03). Additionally, adherence to mask guidance was associated with more acceptance of the COVID-19 vaccine (z = 4.22, *p* = 2.42 × 10^−5^). It is observed that the lower the fear from COVID-19, the higher vaccine acceptance was (χ^2^_(1)_ = 5.39, *p* = 0.02).

We explored the effect of the adjusted background factors on COVID-19 vaccine acceptance by modelling the data using multiple logistic regression entering the variables in a single full model in backward stepwise selection to account for the simultaneous effects of all of them. Table 4 shows the variables that were found to be significant on applying multiple logistic regression, that is, university education (AOR = 1.04, *p* = 0.0306), older pregnancy age (AOR = 6.120, *p* = 0.0097), confidence in vaccine safety (AOR = 3.43, *p* = 0.0001), and efficacy (AOR = 0.36, *p* = 0.0084) had a significant effect on acceptance of vaccination.

## 4. Discussion

The current investigation was carried out among a large sample of pregnant women attending healthcare facilities in Abha, Asir region, Saudi Arabia. Our main and striking finding is that the acceptance rate for COVID-19 vaccination was 93.7% among the participating pregnant women. This finding is different from a lot of other studies reported from various parts of the world. Other studies have reported COVID-19 vaccine acceptance rates ranging from a low of 2.7% in Sudan [21] up to 88% in Australia and New Zealand [22]. If we consider these differences attributable to the varying socio-economic conditions, we also notice a difference in the vaccine acceptance rates across the time-period of the COVID-19 pandemic. During August 2020, when vaccines were not yet developed and their use was not started, only 41% of pregnant women in a survey indicated their willingness to receive the COVID-19 vaccine [23]. Similar to this, a 44.3% vaccine acceptance estimate was reported in a January 2021 survey [24]. In August 2021 survey, there was increased acceptance rate of COVID-19 vaccine (over 60%) [25,26]. More recent surveys from December 2021 onwards have further found better acceptance rates among pregnant women. One study reported a promising 76.6% acceptance rate among pregnant women, surpassing the rate among breastfeeding women (namely, 48.8%) [27]. This could be interpreted as an uptrend in the acceptance of vaccine among pregnant women with time.

The wide spectrum of COVID-19 vaccine uptake indicates that there could be a multitude of factors involved in vaccine acceptance for a new vaccine like COVID-1 [21,22]. Considering the wide variations and noting the high rate of vaccine acceptance in our study, it is critical to identify the factors that can have these influences on vaccine acceptance. Let us try to understand the reasons why vaccine acceptance against COVID-19 is so varied by comparing findings from our study with previously published works.

The uptrend in vaccine acceptance noted between 2021–2022, may be attributed to increasing availability of information related to effectiveness and safety of vaccine. This goes in line with the findings from previous studies which reported that concern about vaccine safety was the major reason for COVID-19 vaccine hesitancy [14]. This is also strengthened by study findings on vaccine safety and effectiveness as a predictors of vaccine acceptance. We will discuss this further after discussing the significant background factors.

Among the background factors, education and gestational age were found to predict COVID-19 vaccine acceptance. We found that university graduates were more likely to accept COVID-19 vaccination among pregnant women. Our findings supported the same tendency for the Saudi public to accept vaccination if they attained higher educational qualification [28]. A similar association was also reported among patients with chronic disease in Saudi Arabia, as better education was linked to higher level of acceptance of COVID-19 vaccine [29]. Obviously, the more educated would have access to more accurate information about vaccine safety and necessity. Similar findings were reported in other studies, notably in Sudan, where husbands’ education was reported to influence vaccine acceptance [21]. Information communicated by health professionals forms an important aspect of health communication, and studies have reported a higher acceptance in women who received vaccine need and importance communication by a health professional [21].

Our study addressed gestational age as a predictor of vaccine acceptance, as many previous studies did not evaluate its potential effect. We found that women with advanced pregnancy were more likely to accept COVID-19 vaccination. This may be related to the communication by physicians of higher safety levels in a more developed fetus, particularly at the last trimester of pregnancy [30]. While studies in Turkey [17] and Iran [31] showed vaccine acceptance in lower gestational age, Blakeway H. et al. in the USA reported a similar finding to ours in their study on COVID-19 vaccination during pregnancy [32].

Among disease related factors, fear of the disease itself is a factor that impresses upon the attitudes towards vaccination. According to our findings, the participant who reported a lesser fear from COVID-19 were more accepting of its vaccination, but this did not become significant when adjusted to the effect of other variables. This finding is opposite to the results from previous studies [9]. However, further research should be directed towards studying any overarching association between corona phobia and vaccine acceptance.

Among the COVID-19 vaccine associated factors, confidence in vaccine safety and efficacy was associated with better acceptance. Numerous studies have shown that vaccine acceptance was associated with confidence in safety and effectiveness [9,33,34]. Concern about vaccine safety remains a ubiquitous barrier, compound by the concern about safety on the developing fetus as found by a previous survey [17]. Vaccine safety to their developing baby would be the top priority for pregnant women in terms of accepting any vaccine to be delivered [35,36]. The initial reports of COVID-19 vaccine safety were not the most reassuring for pregnant women [37], more so than later reports [38], therefore one could relate why the initial uptake was extremely low. Recent research has also indicated that safety comes first and then comes effectiveness in terms of drivers for vaccine uptake among pregnant women [27].

### Strengths and Limitations of the Study

We note many strengths of the current survey. We included a large number of pregnant women with a variety of backgrounds at different pregnancy stages. One significant limitation in the current research is the potential for social desirability bias that could have overestimated the vaccine acceptance rate among our participants [39]. Hence, our results may have overestimated the true prevalence of vaccine acceptance during pregnancy in the region. Our study was done in an urban region and thus it has a high proportion of university educated women, which limits the generalizability of our findings and introduces bias. Nevertheless, the results are promising.

## 5. Conclusions

The acceptance rate for vaccination among pregnant women in Abha, Saudi Arabia, is high. Our results indicate that confidence in vaccine safety and effectiveness was associated with better acceptance. Public health policies should capitalize on such positive attitudes and aim for total coverage of pregnant women with COVID-19 vaccination including booster dosages. Educational campaigns aimed at encouraging women to take the COVD-19 vaccine during pregnancy should emphasize vaccine safety with regard to the fetus along with vaccine effectiveness against COVID-19 infection. Further research into COVID-19 vaccine acceptance should maintain a focus on qualitative design and explore the relationship between acceptance, coronaphobia, and gestational age. In general, identifying attitudes among priority groups will be useful for creating vaccination strategies that increase uptake during the pandemic situations. Specifically, for COVID-19, it is also recommended that research should adopt a longitudinal design and tentatively sample women for calculation of vaccine acceptance rates on monthly basis to evaluate any potential trend.

## Figures and Tables

**Table 1:** Baseline demographics of the study participants and their effect on vaccine acceptance.

Variable	All Participant	COVID-19 Vaccine		*p* Value
		Acceptors	Non Acceptors	
Age in years (Mean ± SD)	29.9 ± 6.92	29.9 ± 5.56	31 ± 7.11	0.299
Number of Children	2.23 ± 2.12	2.17 ± 1.76	3.00 ± 2.55	0.044
Gestational age in weeks	15.1 ± 11.9	15.4 ± 11.7	11.6 ± 11.2	0.036
Occupation				
Employee	166 (29)	157 (94.6)	6 (5.4)	0.001
Housewife	295 (41.6)	271 (91.9)	23 (8.1)	
Student	111 (19.4)	108 (97.3)	3 (2.7)	
Education				
Uneducated	3 (0.5)	2 (66.7)	1 (33.3)	0.001
Primary	8 (1.4)	7 (87.5)	1 (12.5)	
Intermediate	19 (3.3)	14 (74)	5 (26)	
Secondary	110 (19.2)	102 (92.7)	8 (7.3)	
University	420 (73.4)	399 (95)	21 (5.0)	
Postgraduate	12 (2.2)	12 (100)	0 (0)	

**Table 2:** COVID-19 related factors of the study participants and their effect on vaccine acceptance.

Factor	Frequency	COVID-19 Vaccine Acceptance Rate n [%]	*p* Value
Diagnosed with COVID-19	110	108 [98.2%]	0.053
COVID-19 negative effect	96	93 [96.9%]	0.241
COVID-19 related family loss	80	80 [100%]	0.024
COVID-19 related occupational and financial loss	56	53 [94.6%]	0.988
Worry about COVID 19 Quite worried Somewhat worried Neutral Quite unworried Not worried at all	90 200 127 114 41	84 [93%] 189 [94.5%] 116 [91.3%] 108 [94.7%] 39 [95.1%]	0.857
Use of Mask Always Usually Often Rarely Never	325 141 63 22 21	314 [96.6%] 131 [92.9%] 52 [82.5%] 20 [90.9%] 19 [90.5%]	0.002

**Table 3:** COVID-19 vaccine related opinions of the study participants and their effect on vaccine acceptance.

Factor	Freq(n)	COVID-19 Vaccine Acceptance Rate n [%]	*p* Value
Confidence in Vaccine safety Very sure Sure Neutral Unsure Very unsure	184 151 170 43 24	181 [98.4%] 143 [94.7%] 156 [91.8%] 35 [81.4%] 21 [87.5%]	0.016
Confidence in Vaccine efficacy Very sure Sure Neutral Unsure Very unsure	169 176 153 49 25	166 [98.2%] 163 [92.6%] 139 [90.8%] 45 [91.8%] 23 [92%]	0.020
Vaccine importance in general Very important Important Neutral Not important	329 138 85 20	322 [97.9%] 124 [89.9%] 74 [87.1%] 16 [80%]	0.035
COVID-19 vaccine importance Quite important Important Neutral Not important	330 132 85 24	322 [97.6%] 120 [90.9%] 73 [85.9%] 17 [89.5%]	0.032
COVID-19 vaccine fear	471	447 [88.1%]	0.020
Difficulty getting COVID-19 vaccine	61	56 [91.8%]	0.712

**Table 4:** Estimates for the adjusted effects of background factors on COVID-19 vaccine acceptance.

Variable	Adjusted Odds Ratio	95% CI of AOR	*p* Value
Education level	6.120	1.549 to 24.178	0.0056 *
Pregnancy Duration	1.040	1.004 to 1.078	0.0343 *
Confidence In Safety	3.431	1.835 to 6.416	0.0013 *
Confidence In Efficacy	0.364	0.184 to 0.723	0.0084 *

* significant.

## Data Availability

The data presented in this study are available on request from the first author.
